# Transferability of Ligand Field Parameters in a Family of 3d-4f M_2_Ln_2_ Butterfly Single-Molecule Magnets

**DOI:** 10.1021/acs.inorgchem.4c05421

**Published:** 2025-03-17

**Authors:** Julius Mutschler, Thomas Ruppert, Julius Strahringer, Sören Schlittenhardt, Zayan Ahsan Ali, Yan Peng, Christopher E. Anson, Mario Ruben, Annie K. Powell, Oliver Waldmann

**Affiliations:** † Physikalisches Institut, Universität Freiburg, Hermann-Herder-Strasse 3, 79104 Freiburg, Germany; ‡ Institute of Inorganic Chemistry, Karlsruhe Institute of Technology, Kaiserstr. 12, 76131 Karlsruhe, Germany; § Institute of Nanotechnology, Karlsruhe Institute of Technology, Kaiserstr. 12, 76131 Karlsruhe, Germany; ∥ Centre Européen de Sciences Quantiques (CESQ), Institut de Science et d’Ingénierie Supramoléculaires (ISIS), 8 Allée Gaspard Monge, BP 70028, 67083 Strasbourg Cedex, France; ⊥ Institute of Quantum Materials and Technologies, Karlsruhe Institute of Technology, Kaiserstr. 12, 76131 Karlsruhe, Germany

## Abstract

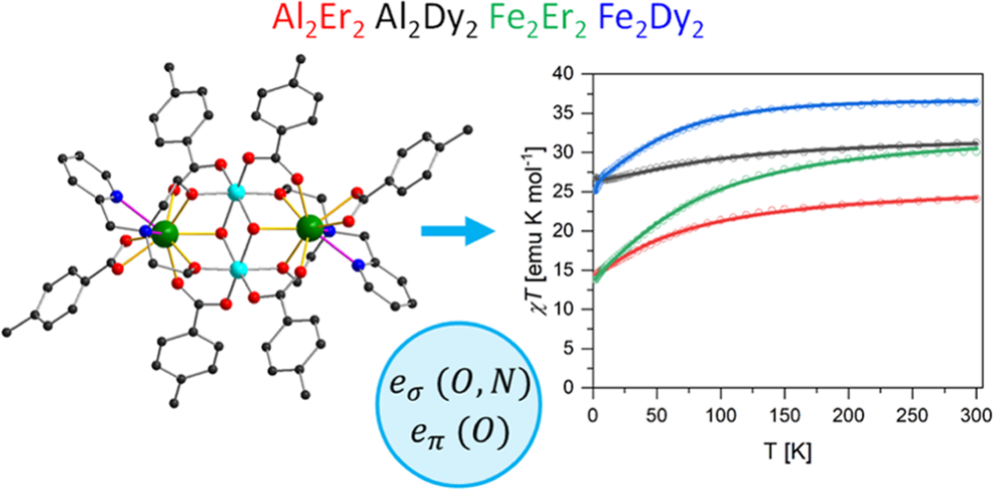

Lanthanide-based single-molecule magnets are attractive candidates for applications in quantum information processing or data storage. The low symmetry ligand field environment often encountered in these complexes, combined with a sparsity of information in the common magnetic susceptibility and magnetization data recorded on powder samples, leads to massive overparametrization. This limits the application of ligand field models, such as the point charge or angular overlap models, for describing the data. In this work, the radical approach is taken to consider these models as black boxes, whereby they suspend their chemical or physical significance. Instead, the transferability of parameters across a series of structurally related compounds is enforced to achieve a reduced parametrization. This methodology is applied to four members of the isostructural series of [M_2_^III^Ln_2_^III^(μ_3_–OH)_2_(pmide)_2_(p-Me-PhCO_2_)_6_]·2MeCN (pmideH_2_ = *N*-(2-pyridylmethyl)-iminodiethanol) butterfly complexes, with M = Al, Fe, and Ln = Er, Dy. It is shown that the powder magnetic susceptibility and magnetization data can be described simultaneously with remarkable accuracy using only two parameters for characterizing the lanthanide ligand fields in all four compounds. Interestingly, the resulting parametrization and its values lie within the range of chemical intuition despite the black box procedure used to obtain them. Implications of this approach are discussed.

## Abstract 2

The ligand field in 4f-based single-molecule magnets often lacks symmetry, which leads to massive overparametrization. In this work, the radical approach is taken to consider ligand field models like the Angular Overlap Model as black boxes, suspending their chemical significance. Instead, the transferability of parameters is enforced. For a series of four 3d-4f butterfly compounds, this allowed us to describe their magnetic data with remarkable accuracy using only two parameters for characterizing the ligand field.

## Introduction

Lanthanide-containing magnetic molecules have attracted enormous attention in the recent decade due to their potential for unprecedented magnetic behaviors, such as single-molecule magnetism (SMM) at liquid nitrogen temperatures and properties pertinent to quantum information technologies.^1−10^ While the magnetism of lanthanide ions was extensively studied in the latter half of the 20th century, with fundamental theories and models well established,^11^ the specific circumstances realized in many of the contemporary 4f-based magnetic molecules pose new challenges for both theory and experiment.^12−19^

A key novel aspect in many of these molecules is that the ligand field environment of the lanthanide ions often exhibits very low symmetry or lacks any symmetry elements entirely. As a result, all ligand field parameters *B*_*kq*_, which occur in the decomposition of the *J* multiplet spin Hamiltonian in terms of Stevens operators Ô_*kq*_ (*k* = 2,4,··· ≤ 2*J*, *q* = −*k*,···,*k*, the *k* = 0 term is ignored as it only contributes a constant),^20−24^ must be considered in experimental modeling since there is no a priori reason to ignore or correlate any of these parameters (*J* is the free ion total angular momentum). This work is concerned with the magnetic properties of the molecules, and a discussion in terms of the ground state *J* multiplet as determined by Hund’s rules is thus appropriate (*J* = 15/2 for Er^III^ and Dy^III^).^25^ In the case of Dy^III^ or Er^III^ ions, the theory demands a staggering amount of 119 ligand field parameters (since *k* = 2,4,···,14). This number is commonly reduced to 27 parameters corresponding to orders *k* = 2,4,6, which is the number of parameters obtained in first-order perturbation theory treatments.^20^ However, this reduction is somewhat artificial, as it is well-known that effects such as configuration interaction and covalency can make higher-order terms more significant than expected in first-order perturbation theory.^20^ Some (phenomenological) theories attempt to model these effects at the cost of introducing dozens of additional parameters. For instance, a common model for lanthanide-free ions involves 20 atomic parameters such as the Racah or Condon-Shortly parameters, spin-orbit coupling constant, Tree parameters, and so on.^26−30^ The situation is thus not fundamentally improved with regard to the number of parameters.

High-level theoretical methods such as Complete Active Space Self Consistent Field (CASSCF) *ab initio* techniques have made tremendous progress in recent times, and state-of-the-art codes can provide quantitative results for ligand field parameters of lanthanide ions.^31−34^ However, it appears fair to say that these techniques have not yet reached the full quantitative predictive level.^35^ They are also involved and not for routine application by experimental chemists and physicists. Therefore, phenomenological models developed decades ago, such as the Point Charge Model (PCM), the superposition model, or the Angular Overlap Model (AOM), have seen renewed interest and have been extended in various ways to enhance their predictive capabilities.^20,23,36−42^ For example, PCMs were proposed which consider displaced positions of the ligand charges.^36,42^ Efforts also exist to link phenomenological AOM and *ab initio* techniques.^43−45^ Such models could be appealing for routine ligand field analyses, as they incorporate information about the ligand environment, typically by using the atomic positions from experimental X-ray crystal structure analyses, which suggests that they might require significantly fewer phenomenological parameters. The parameters in these models are also often considered to be more chemically intuitive, potentially making it easier to guess appropriate parameter values.^46^ Moreover, the ligand field parameters *B*_*kq*_ depend only on the metal ion and the nature and position of the ligands and should in principle be transferable to some extent within structurally similar molecules.^47,48^ However, these promises have not materialized in practice, as partially evidenced, e.g., by the relatively few applications in the recent decade.^41,49^

Experimentally, the large number of parameters required in ligand field models necessitates a substantial amount of feature-rich data to accurately determine these parameters. However, the data obtained from measurements of the temperature-dependent magnetic susceptibility and field-dependent magnetization curves at low temperatures on powder samples, which are the most widely employed experimental techniques for accessing the magnetic properties of these clusters, are often relatively featureless. In many cases, the data from these techniques form the backbone for the analysis of magnetism in these compounds.^50^ With up to 27 parameters per lanthanide ion and potentially more for also describing intramolecular exchange interactions in polynuclear complexes, the task of determining them from the relatively featureless data becomes hopeless.

In the basic ligand field theories, the transferability of ligand field parameters is reflected in a separation of the ligand field parameters into two factors: one depending on the type of lanthanide ion and the other describing the ligand field environment.^51^ Chemically, it is often feasible to substitute the lanthanide centers, giving rise to a series of isostructural compounds. It is thus favorable to study several members of such a family in order to increase the amount of experimental information available.^50^ However, the effectiveness of this approach, in terms of the number of parameters required for characterizing the ligand field, hinges on how well transferability is actually realized in a given family of compounds.

Recent studies on transferability include refs (52,53), where each ligand field parameter was described by a linear function of the atomic number of the lanthanides. This approach was also repeated in later works.^54^ Along similar lines, in ref (55), it was assumed that the ligand field parameters depend linearly on the number of 4f electrons. While successful to some extent, this parametrization scheme still leaves two parameters to be determined for each ligand field parameter *B*_*kq*_, i.e., 54 parameters in the general case.

Given the challenges in modeling data sets like powder magnetic susceptibility and magnetization curves for various compounds within a family, we opted for a drastic approach. We chose to disregard any a priori physical or chemical relevance of the ligand field parameters and instead tackle the challenge from a purely information theoretical point of view. The objective is then to design a mathematical device while ignoring its physical or chemical interpretation, which is capable of accurately reproducing all available experimental data, such as the magnetic susceptibility and magnetization curves, using a minimal number of input parameters.

The implementation of such a black box device naturally lends itself to statistical and data-oriented techniques, such as machine learning. In this work, however, a different approach is taken that utilizes the common ligand field theories but in a black box manner. The spin Hamiltonian is utilized as a device to generate physically reasonable magnetic susceptibility and magnetization curves. It is prepended with a black box, which maps a few input parameters to the 27 ligand field parameters for the different lanthanide ions as required for the spin Hamiltonian, accomplishing the desired parameter reduction. Other parameters in the spin Hamiltonian, such as exchange coupling constants, are supplied directly to the model. The black box prepending the spin Hamiltonian could be powered by various methods, including machine learning algorithms, but in this study, the mathematical equations of the PCM or AOM are employed. The resulting mathematical device is similar to previous ligand field analyses using PCM or AOM with the important difference that the input parameters are not given a priori chemical or physical significance. Chemical or physical bias is thus lacking, which allows for unconstrained mapping. The constraint is instead shifted from chemical/physical input parameters toward requiring transferability of the ligand field parameters within a family of compounds. The convergence of this approach to chemically or physically meaningful parameters is neither obvious a priori nor intended, but the usefulness of the approach can easily be judged by its success in simultaneously modeling the various experimental data with few input parameters.

**Figure 1:**
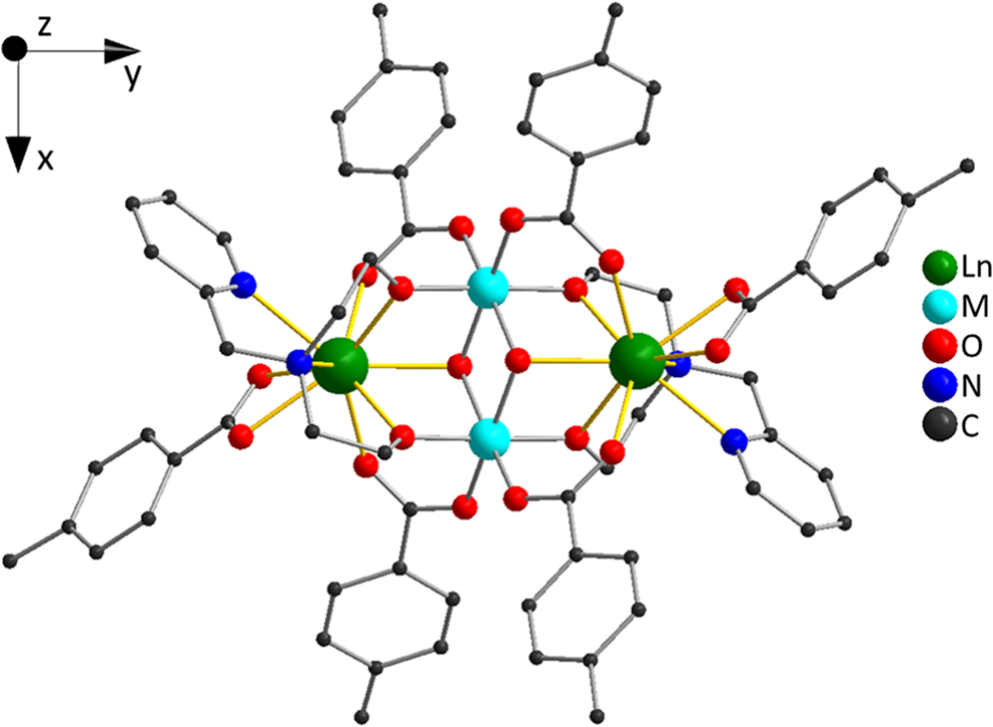
Molecular structure of the M_2_Ln_2_ butterflies. The legend on the right gives the color scheme for the atoms. For clarity, the bonds from the Ln^3+^ ions to the ligand atoms in the first coordination sphere are highlighted in yellow. H atoms are omitted. The top left corner shows the coordinate system used in the magnetic model.

In this work, the magnetism of four members of the family of molecules [M_2_^III^Ln_2_^III^(μ_3_–OH)_2_(pmide)_2_(p-Me-PhCO_2_)_6_]·2MeCN (pmideH_2_ = *N*-(2-pyridylmethyl)-iminodiethanol) is investigated, with M = Al or Fe and Ln = Er or Dy: Al_2_Er_2_ (**1**), Al_2_Dy_2_ (**2**), Fe_2_Er_2_ (**3**), and Fe_2_Dy_2_ (**4**).^56,57^ These molecules exhibit a butterfly structure, where the 3d metal ions form the body and the 4f ions form the wings of the butterfly (Figure 1). Al^III^ is diamagnetic, and the two compounds Al_2_Er_2_ (**1**) and Al_2_Dy_2_ (**2**) thus provide insight into the 4f single-ion magnetism, undisturbed by the effects from the 3d metal ions. The magnetic susceptibility and magnetization curves for these compounds have been previously published.^56,57^ However, these data were remeasured as part of this work. Successful modeling was found to be critically dependent on the quality of the experimental data; ensuring reproducibility and consistency in the measurement protocol and care in avoiding the typical experimental inaccuracies were identified as key factors. This observation should not be surprising since the featureless nature of the data implies that minor experimental inaccuracies can lead to significant changes in the model parameters. It emphasizes, however, the importance of subjecting only carefully measured data of this kind to theoretical comparison. It will be demonstrated that the AOM black box approach described above allows us to reproduce the experimental data of all four compounds with high accuracy, utilizing only two parameters to characterize the ligand field of the lanthanide ions in the four compounds. Our results presented here provide a remarkable experimental example of using transferability to achieve a reduced parameterization of ligand field.

## Experimental Methods

Compounds **1**–**4** were synthesized following literature procedures.^56,57^ The magnetic properties were recorded on powder samples using an MPMS SQUID VSM from Quantum Design. The temperature-dependent magnetic susceptibility χ(*T*) curves were obtained from measurements at an applied magnetic field of 0.1 T. The magnetization curves as a function of applied magnetic field *M*(*B*) were measured at temperatures of 2, 3, 4, and 5 K, and the maximum magnetic field was 7 T. In order to ensure highly reproducible and accurate magnetic data, the following procedure was applied to each compound: The samples were mixed with eicosane to prevent alignment of the microcrystallites in the applied magnetic field. The measurement protocol was as follows: (1) apply 0.1 T and measure χ(*T*) while cooling down from 300 to 2 K. (2) Measure χ(*T*) while heating up from 2 to 300 K. (3) Measure χ(*T*) while cooling down from 300 to 2 K. (4) Apply 0 T and measure *M*(*B*) by varying field from 0 to 7 T at temperatures of 2, 3, 4, and 5 K. (5) Apply 0.1 T and measure χ(*T*) while heating up from 2 to 300 K. Before each step, a waiting period of 30 min was inserted. The comparison of the data from runs 1 and 2 permits one to check whether the temperature sweep rate was appropriate, and comparing these data with the data from run 5 permits one to check for effects of reorientations or alignments in the sample. All data were corrected for diamagnetic contributions from the sample holder and the eicosane. It should be noted that the diamagnetic correction could be slightly inaccurate, which could be accounted for in χ(*T*) simulations by adding correction δχ_0_ to the simulated curves. Furthermore, the sample mass might be overestimated due to the potential loss of material when mixing with eicosane. In addition, the molecular mass might not be accurately known due to, for example, the loss of solvent molecules. These effects could be accounted for in simulations by multiplying the simulated curves with mass correction factor *m*_*c*_. For reference, susceptibility curves were also recorded on powder samples that were not prepared with eicosane, yielding more accurate sample masses. The magnetic susceptibility curves are shown as χ*T* vs *T* curves. In data plots, the experimental data are generally represented by symbols, whereas simulated data are generally represented by lines.

## Theoretical Methods

Using the spin Hamiltonian approach, the compounds Al_2_Er_2_ (**1**) and Al_2_Dy_2_ (**2**) were modeled as dimers of two lanthanide ions with total angular momentum *J* = 15/2, with a Ln^III^–Ln^III^ Heisenberg exchange coupling of strength *J*_Ln–Ln_, single-ion magnetic anisotropy represented by the 27 Stevens operators of up to sixth order (*k* = 2,4,6), and a Zeeman term with Landé factor *g*_Ln_ (*g*_Er_ = 1.2, *g*_Dy_ = 1.33 for free ions). The Ln^III^–Ln^III^ exchange coupling can be expected to be small but was found to be relevant. For instance, in Al_2_Dy_2_ (**2**), it accounts for an upturn in the χ*T* curve at low temperatures. In addition to the exchange interactions, dipolar magnetic interactions can also be expected. However, as tested by explicit simulations, their effect is indistinguishable from exchange interactions with regard to the magnetic data presented in this work and thus need not be explicitly introduced. The reported values for *J*_Ln–Ln_ account for the combined effect of exchange and dipolar interactions. The spin Hamiltonian reads
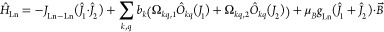
1Here, the lanthanide ligand field is parametrized by the “bare” ligand field parameters Ω_*kq,j*_ (*j* = 1,2 for the two Ln^III^ ions in the cluster), which are related to the common ligand field parameters *B*_*kq,j*_ as *B*_*kq,j*_ = *b*_*k*_ Ω_*kq,j*_, with *b*_*k*_ = θ_*k*_⟨*r*^*k*^⟩ or *b*_*k*_ = θ_*k*_ for the PCM and AOM, respectively. θ_*k*_ is the Stevens factor and ⟨*r*^*k*^⟩ the average radial function of the *k*-th order. The other symbols in the equation have their usual meaning. In the PCM and AOM, the bare ligand field parameters Ω_*kq,j*_ are expected to be independent of the lanthanide central ion and solely determined by the ligand environment and should therefore be directly comparable across the different compounds. In contrast, the factors θ_*k*_ and ⟨*r*^*k*^⟩ depend on the central lanthanide ion and are not free parameters.

For compounds Fe_2_Er_2_ (**3**) and Fe_2_Dy_2_ (**4**), the spin Hamiltonian was extended by Fe^III^–Fe^III^ and Fe^III^-Ln^III^ Heisenberg exchange interactions of strengths *J*_Fe–Fe_ and *J*_Fe–Ln_, single-ion anisotropy terms *D* and *E* to describe the anisotropy of the Fe^III^ ions and a Zeeman term with *g* factor *g*_Fe_ (*S* = 5/2 for high-spin Fe^III^). The spin Hamiltonian reads
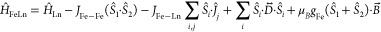
2where *i* = 1,2 indexes the two Fe^III^ ions in the cluster (and *j* = 1,2 for the two Ln^III^ ions as before), and .

The lanthanide ions in the considered compounds are each 9-fold coordinated, with seven oxygen and two nitrogen atoms in the first coordination sphere. In the PCM, these are represented by fictious charges *q*_*n*_ (*n* = 1,···,9) located at the positions of the atoms as determined from the experimental X-ray crystal structures. The model was extended in various ways, e.g., by introducing a radial scaling factor *R*_*s*_, which multiplies the ligand distances and allows variation of the ligand field strength. In the AOM, each ligand is characterized by seven angular overlap parameters, *e*_σ_, *e*_π,*c*_, *e*_π,*s*_, *e*_δ,*c*_, *e*_δ,*s*_, *e*_φ,*c*_, and *e*_φ,*s*_, or bond parameters in short, which have the usual meaning (see also Figure S1).^38^ The directions (polar and azimuthal angles θ and ϕ) for each ligand were again determined from the experimental X-ray crystal structures.

In the PCM, the bare ligand field parameters for one lanthanide ion are calculated as^21^
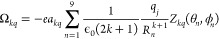
3Here, *R*_*n*_ is the distance and θ_*n*_, ϕ_*n*_ are the polar and azimuthal angles of the *n*-th ligand. The factors *a*_*kq*_ account for the unsystematic normalization of the Stevens operators as compared to spherical harmonics,^21^ and the other symbols have their usual meaning. Following ref (38), the equation for calculating the bare ligand field parameters in the AOM can be written as
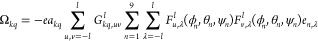
4where *l* = 3 for 4f ions, and *F*_*u*,λ_^*l*^(ϕ,θ,ψ) are the angular overlap integrals (ψ = 0 in this work). The factors *e*_*n*,λ_ (with λ = −*l*,···,*l*) are the bond parameters for each ligand, which are related to those introduced in the above as *e*_σ_ = *e*_λ=0_, *e*_π,*c*/*s*_ = *e*_λ=±1_, *e*_δ,*c*/*s*_ = *e*_λ=±2_, *e*_φ,*c*/*s*_ = *e*_λ=±3_. The *G*_*kq,uv*_^*l*^ are calculated as
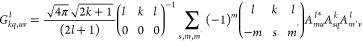
5where the range of the indices is *m*, *m*′,*u*,*v*,λ ∈ [−*l*,*l*] and *s*, *q* ∈ [−*k*,*k*]. The matrices *A⃡*^*k*^ describe the transformation from spherical to tesseral harmonics, *Z*_*kq*_ = ∑_*q*′_*A*_*qq′*_^*k*^*Y*_*kq*_, and the (:::) brackets are Wigner 3j symbols.

The simulation of the magnetic susceptibility and magnetization curves consists of numerically diagonalizing the Hamiltonian matrix and applying standard equations from quantum statistics using an in-house code. The susceptibility is calculated for the three magnetic field directions along the *x*, *y*, and *z* axes, and the powder susceptibility is then obtained as . These simulations are readily possible on a modern PC, even for the compounds Fe_2_Er_2_ (**3**) and Fe_2_Dy_2_ (**4**), which exhibit Hilbert space dimensions of 9216. However, simulating the powder magnetization curves is significantly more time-consuming because a powder average must be performed for each magnetic field value. Permutation symmetry was utilized in order to block diagonalize the Hamiltonian matrix, which reduced computation time by ca. a factor 10 using the Klein four-group.^58,59^ Yet, for these two compounds, only simulation of the magnetization curves was achieved, whereas fits to the magnetization curves were found to be prohibitive.

The various variants of AOMs start from a specific parametrization of the single-electron coulomb interaction potential.^60^ In the simplest variant, outlined for instance in ref (38), it is directly projected into the space of the ground state *J* multiplet of the lanthanide ion. This corresponds to the first-order perturbation theory. In more sophisticated models, effects such as configuration interaction are taken into account, and more details of the atomic structure and the bonds need to be modeled. As a result, further parameters enter in these theories, such as the aforementioned atomic Racah or Slater–Condon parameters, spin-orbit coupling constant, reduction factors, and so on. In this work, the simplest AOM of ref (38) is applied for two reasons. First, it can be seen as an effective Hamiltonian at the single-electron level. That is, the result of any higher-level theory for the spin Hamiltonian in the *J* multiplet space can always be projected back to the single-electron space (using the inversions of eqs 4 and 5) and be cast in terms of the AOM model and parameters. The model may lose accuracy, and the parameters may lose their chemical/physical interpretability, which, however, leads to the second reason. As pointed out before, what we are looking for is only a mathematical device for successful parameter reduction. A possible lack of chemical or physical interpretability of the model is thus irrelevant to the purpose and spirit of this work.

The molecules are slightly noncentrosymmetric, and the ligand environments for the two lanthanide ions within a molecule are not exactly identical. In the PCM and AOM, this is accounted for by using the experimental X-ray atomic positions. For modeling in terms of the bare ligand field parameters, the difference is small enough to be negligible, and Ω_*kq*,1_ = Ω_*kq*,2_ = Ω_*kq*_ was set. The coordinate frame used in this work (see Figure 1) is chosen such that the *y*-axis is oriented along the connection of the two lanthanide ions in the cluster and the *z-*axis is perpendicular to the plane spanned by the four metal ions. The *x*-axis is then nearly, but not exactly, parallel to the Fe^III^ ion connection line. The origin of the coordinate frame is placed in the center of the molecule, as determined from the least distance from the Ln^III^–Ln^III^ and Fe^III^–Fe^III^ connection lines. The employed ligand positions are listed in Table S1.

## Results and Discussion

### Magnetic Data

**Figure 2:**
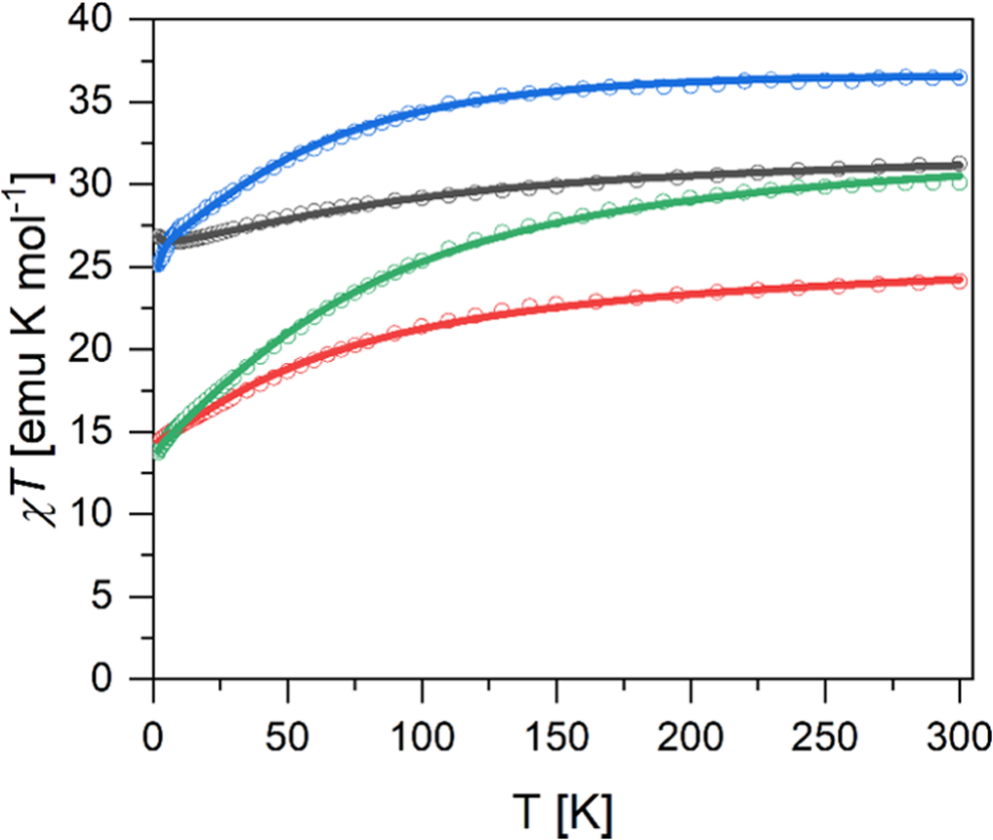
Experimental magnetic susceptibility *χT* curves (open circles) of Fe_2_Dy_2_ (**4**) (blue), Al_2_Dy_2_ (**2**) (black), Fe_2_Er_2_ (**3**) (green), and Al_2_Er_2_ (**1**) (red). The solid lines represent the corresponding fits by using the model and parameters stated in the text.

**Figure 3:**
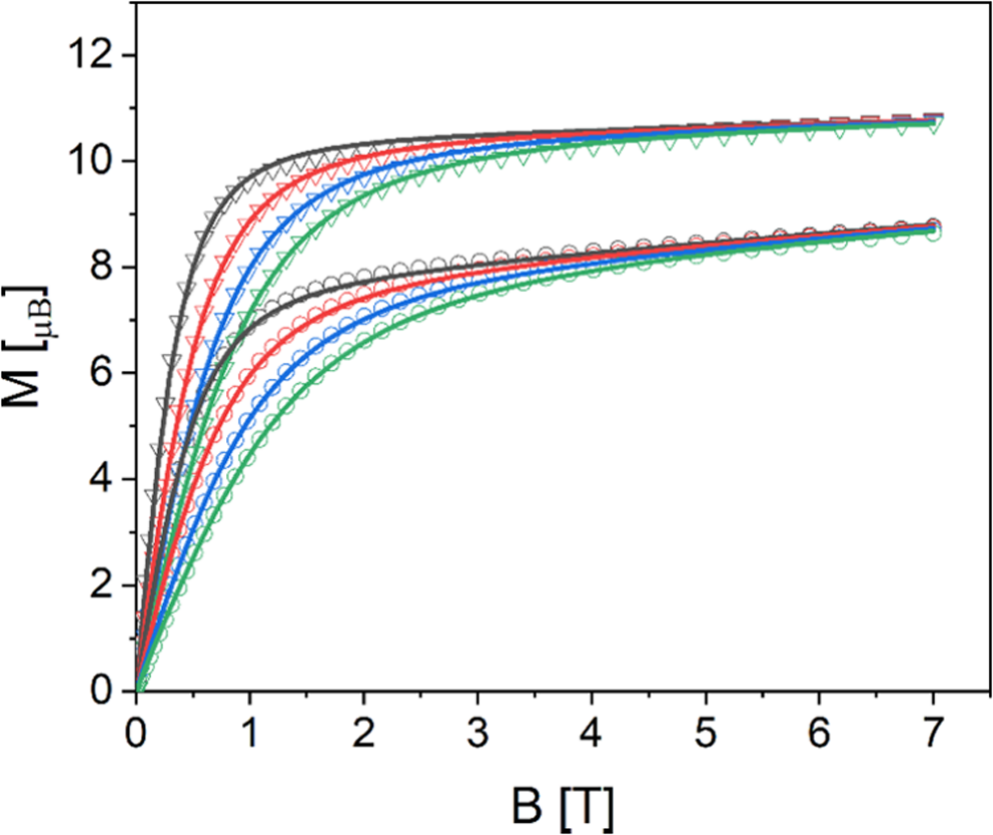
Experimental *M*(*B*) curves of Al_2_Er_2_ (**1**) (open circles) and Al_2_Dy_2_ (**2**) (open triangles) at temperatures of 2 K (black), 3 K (red), 4 K (blue), and 5 K (green). The solid lines represent the corresponding fits using the model and parameters stated in the text.

**Figure 4:**
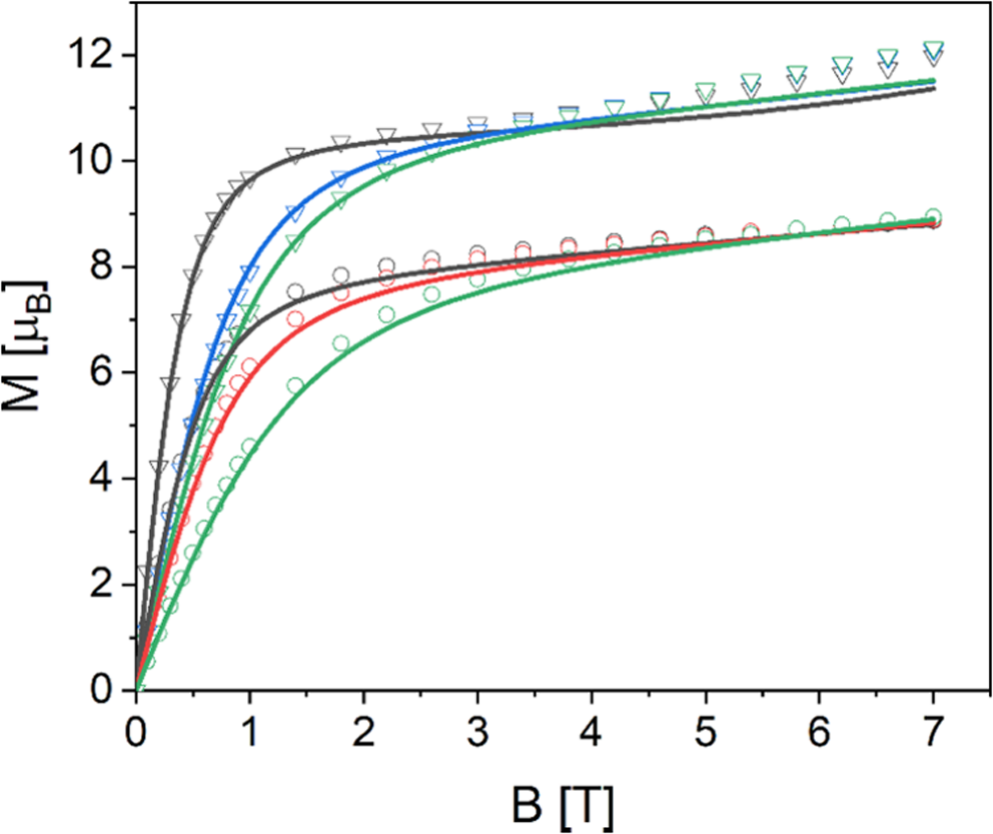
Experimental *M*(*B*) curves of Fe_2_Er_2_ (**3**) (open circles) and Fe_2_Dy_2_ (**4**) (open triangles) at temperatures of 2 K (black), 3 K (red), and 5 K (green) for (**3**) and 2 K (black), 4 K (blue), and 5 K (green) for (**4**). The solid lines represent the corresponding fits using the model and parameters stated in the text.

While the magnetic data for compounds **1**–**4** were remeasured as part of this work and show some differences from the published data, the overall behavior remains consistent with previous reports.^56,57^ Therefore, the data are only briefly described here with regard to their main features. For each compound, the χ*T* curve shows the expected value at room temperature and a downturn toward lower temperatures, which is typical for lanthanide-containing molecules due to the thermal depopulation of the lanthanide ligand field states (see Figure 2). In the compound Al_2_Dy_2_ (**2**), an upturn is observed at the lowest temperatures, indicating the presence of weak ferromagnetic interactions in the cluster. A very weak upturn is also observed for Fe_2_Er_2_ (**3**). For Fe_2_Dy_2_ (**4**), the general downward trend with decreasing temperatures is stronger at the lowest temperatures, possibly indicating the presence of predominantly antiferromagnetic interactions. The *M*(*B*) curves also show the expected magnetic field and temperature dependencies for Al_2_Er_2_ (**1**) and Al_2_Dy_2_ (**2**) (see Figure 3). The magnetic field curves first increase linearly with the magnetic field to approach a flat plateau at higher fields, indicating a magnetic ground state that is energetically well separated from the higher-lying levels. In Fe_2_Er_2_ (**3**) and Fe_2_Dy_2_ (**4**), the overall behavior is similar, except that at high fields, the curves for different temperatures cross, indicating a nearby level crossing in both compounds (see Figure 4). The magnetization curves cross at ca. 4.1 and 5.8 T in Fe_2_Dy_2_ (**4**) and Fe_2_Er_2_ (**3**), respectively.

### Ligand Field Analysis for Al_2_Er_2_ (**1**) and Al_2_Dy_2_ (**2**)

The ligand field parameters for compounds Al_2_Er_2_ (**1**) and Al_2_Dy_2_ (**2**) were determined by simultaneously fitting all data, specifically the χ*T* and *M*(*B*) curves for both compounds, to one model. The fitting was accomplished by calculating the χ^2^ landscape as a grid of data points in a suitable parameter range and looking for the minima. Here, χ^2^ was defined as usual as the squared deviation of simulated and experimental data points, summed over all data points.^61,62^ Least-squares fitting routines such as Levenberg–Marquardt^62^ could also have been employed for this part of the work, but the χ^2^ landscapes were found to be particularly insightful. They also helped us to ensure that all of the local minima were identified. It was found useful to calculate the χ^2^ landscapes for each data set and each compound independently, yielding χ_χ*T*_^2^(**1**), χ_*M*_^2^(**1**), χ_χ*T*_^2^(**2**), and χ_*M*_^2^(**2**) landscapes. The total χ^2^ for all data of both compounds was then obtained as χ^2^ = χ_χ*T*_^2^(**1**) + χ_*M*_^2^(**1**) + χ_χ*T*_^2^(**2**) + χ_*M*_^2^(**2**). The susceptibility and magnetization contributions in this equation could be adjusted by weight factors to change the relative emphasis, but it was found by explicit checks that this does not alter our conclusions. The experimental data was reduced to 51 and 32 data points for calculating χ_χ*T*_^2^ and χ_*M*_^2^, respectively. In the model, the exchange couplings *J*_Dy–Dy_ and *J*_Er–Er_ were assumed as independent free parameters, but the bare ligand field parameters were linked, i.e., assumed to be equal for both compounds (but not equal for the two lanthanide ions within a cluster in the PCMs and AOMs). Through this linking of the bare ligand field parameters, their transferability is enforced in the modeling.

In a first fit, the bare ligand field parameters were all set to zero, except Ω_20,1_ = Ω_20,2_ = Ω_20_ and Ω_22,1_ = Ω_22,2_ = Ω_22_. This corresponds to a rhombic model for lanthanide single-ion anisotropy. The model thus consisted of four magnetic parameters (*J*_Dy–Dy_, *J*_Er–Er_, Ω_20_, Ω_22_), and also the effect of the experimental uncertainties δχ_0_ and *m*_*c*_ was investigated. A reasonable fit could not be found (the χ^2^ plots for a representative example are shown in Figure S2). A sufficiently large parameter space was scanned to ensure that no minimum was overlooked. Accordingly, the Levenberg–Marquardt least-squares fitting algorithm was employed to identify reasonable minima using extended models, which introduced more and more bare ligand field parameters as additional magnetic parameters. However, this approach either did not produce satisfying fits or resulted in issues with overparametrization and was thus not further explored.

In the next step, the PCM model was employed in order to set the bare ligand field parameters. In this model, the magnetic parameters were the ligand charges *q*_*n*_ and the couplings *J*_Dy–Dy_ and *J*_Er–Er_. As before, the effect of δχ_0_ and *m*_*c*_ was also explored. In order to reduce the number of parameters, only two different values for the charges were considered, and various combinations and variations were explored. The considered set of different parametrizations included two that were chemically motivated. In one parametrization, the charges of the seven oxygen atoms were set to *q*(*O*) and those of the two nitrogen atoms to *q*(*N*). In another parametrization, inspired by the formal Lewis charges of the ligands, the charges of the nitrogen atoms were set to zero, those of the carboxylate oxygens to *q*_c_(*O*), and those of the alkoxo and hydroxo oxygens to *q*_ah_(*O*). Furthermore, fits were performed with the additional introduction of the radial scaling factor *R*_*s*_. A satisfactory fit that simultaneously describes all data could not be found (exemplary χ^2^ plots are provided in Figure S3).

Lastly, fits using the AOM model for setting the bare ligand field parameters were explored. In this model, the magnetic parameters were the bond strengths *e*_λ_, the couplings *J*_Dy–Dy_ and *J*_Er–Er_, and δχ_0_ and *m*_*c*_ were also considered. In some fits, the Landé *g*_Ln_ factors were also allowed to vary in order to test the stability and robustness of the fit. In order to reduce the number of parameters, only two different values for the bond strengths were considered in various combinations. Of these different parametrizations, the results of the two best performing will be discussed here. In the first parametrization, all nine bonds were assumed to be of pure σ character (*e*_λ_ = 0 for λ ≠ 0) and to be of equal strength *e*_σ_(*O*) and *e*_σ_(*N*) for the oxygen and nitrogen atoms, respectively. Although it was not the deciding factor in its selection, this parametrization may be motivated by the well-known different positions of the oxygen and nitrogen ligands in the spectrochemical series.^63^ In this parametrization, five local minima were obtained (see Figure S4). They reproduce the magnetic data for Al_2_Er_2_ (**1**) and the magnetization curves for Al_2_Dy_2_ (**2**) quite well, but the χ*T* curve for Al_2_Dy_2_ (**2**) is not satisfactorily described for all minima. Furthermore, for four of the five minima, one of the bonding parameters *e*_σ_(*O*) or *e*_σ_(*N*) is obtained as negative; i.e., only for one of the five minima are they both positive. Since the AOM model is regarded in this work as a purely mathematical device for parameter reduction, negative σ bond strengths are not of concern per se but are clearly in contradiction with chemical expectation, i.e., the electron donor property of complex σ bonds.^46^

**Figure 5:**
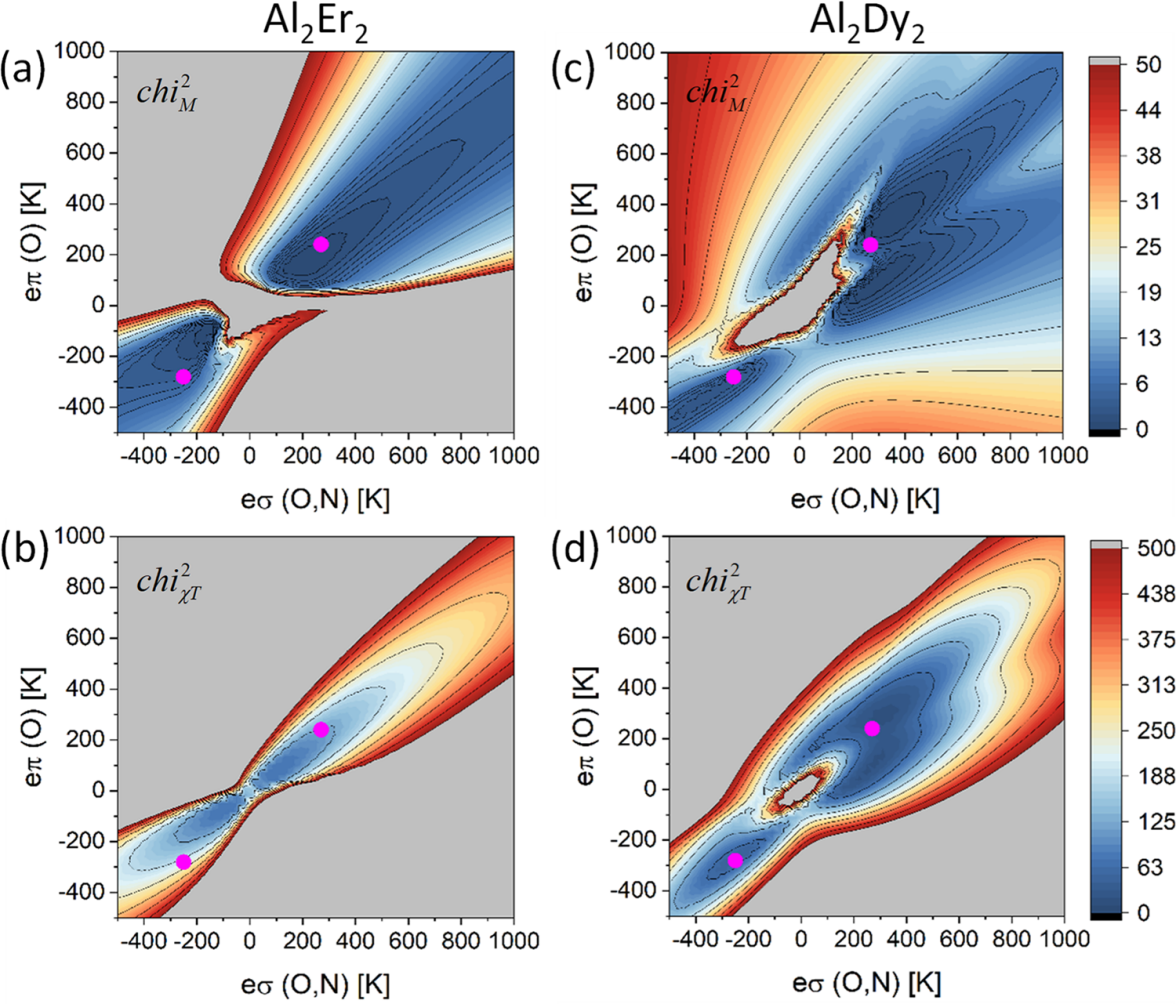
χ^2^ plots for the AOM using *e*_σ_(*O,N*) and *e*_π_(*O*) parametrization. The coupling constants are *J*_Dy–Dy_ = 6 mK and *J*_Er–Er_ = 2 mK. (a) χ_*M*_^2^(**2**), (b) χ_χ*T*_^2^(**2**), (c) χ_*M*_^2^(**1**), and (d) χ_χ*T*_^2^(**1**). The color maps are from blue to red with a range [0, 50] for the two magnetization grids and a range [0, 500] for the two susceptibility grids. The pink dots mark the two local minima with *e*_σ_(*O,N*) = 270 K, *e*_π_(*O*) = 240 K and *e*_σ_(*O,N*) = *–*250 K, *e*_π_(*O*) = *–*280 K.

The best result was obtained with a parametrization where the σ bond strengths were set to equal value for all ligands, i.e., *e*_σ_ = *e*_σ_(*O*, *N*) for all nine ligands, and where a π character was introduced for the oxygen atoms, i.e., *e*_π,*c*_ = *e*_π,*s*_ = *e*_π_(*O*) for the seven oxygen ligands. This parametrization may be motivated by the π-donor character of typical oxygen ligands.^63^ All other bond strengths were set to zero. This yielded two minima, at *e*_σ_(*O,N*) = 270 K, *e*_π_(*O*) = 240 K (χ^2^ = 144) and *e*_σ_(*O*, *N*) = −250 K, *e*_π_(*O*) = −280 K (χ^2^ = 226), see Figure 5. Inspection of the individual plots for χ_χ*T*_^2^(**1**), χ_*M*_^2^(**1**), χ_χ*T*_^2^(**2**), and χ_*M*_^2^(**2**) shows that these two minima do not match the local minima for each of the data sets individually. That is, for each data set, more accurate fits could be obtained. However, both minima are close to the local minima in each individual plot, giving the overall best-fit property. Combining the data also helped reduce the number of possible solutions. For instance, in the χ_χ*T*_^2^(**1**) plot, three local minima are visible (and also in the χ_*M*_^2^(**1**) plot), but one of them is disfavored by the fact that it is in a high χ^2^ area for the data of compound Al_2_Dy_2_ (**2**). The positive bond strengths *e*_σ_(*O*, *N*) and *e*_π_(*O*) for one of the minima bodes well with the expected donor character of σ bonds and π-donor character of carboxylate and alkoxide groups.^63^ For this reason, the minimum with negative bond strengths is not further considered.

In order to refine the fit and test robustness and stability, Levenberg–Marquardt fitting was employed with the above parameter values as the starting values. The model was extended in various ways by adding more free parameters, which gave insight into the robustness/stability of the results. For instance, extending the model by allowing the Landé *g*_Ln_ factors to vary and introducing δχ_0_(**1**) and δχ_0_(**2**) parameters to account for slight inaccuracies in the diamagnetic corrections of the data sets for Al_2_Er_2_ (**1**) and Al_2_Dy_2_ (**2**) (*m*_*c*_ = 1 in these fits) yielded the best-fit result *e*_σ_(*O*, *N*) = 442(5) K, *e*_π_(*O*) = 416(4) K, *J*_Dy–Dy_ = 1.0(1) mK, *J*_Er–Er_ = 1(2) mK, *g*_Dy_ = 1.38(1), *g*_Er_ = 1.22(1), δχ_0_(**1**) = 0.0034(2) emu mol^–1^ and δχ_0_(**2**) = 0.0036(2) emu mol^–1^. The Landé *g*_Ln_ factors are obtained very satisfactorily, within a few percent of the expected values, and the diamagnetic corrections are also found to be very small. This indicates the stability and robustness of the model. The values for the bond strengths come out substantially larger, however, as compared to the results from the χ^2^ landscape analysis, but the ratio *e*_π_(*O*)/*e*_σ_(*O,N*) is essentially preserved. This suggests that the absolute strength of the ligand field cannot be very precisely determined from the information contained in the experimental data, while the anisotropy generated by the ligand field model is robust. The resulting ligand field parameters are given in Tables S2 and S3. The coupling constant *J*_Dy–Dy_ is very small and ferromagnetic but is determined with significance. It is, in fact, responsible for the upturn in the χ*T* curve of Al_2_Dy_2_ (**2**) and is therefore physically significant. In contrast, the coupling constant *J*_Er–Er_ should be considered as not significant.

In passing, it is mentioned that explicitly including dipolar interactions in the model yielded best-fit values *e*_σ_(*O,N*) = 453(3) K, *e*_π_(*O*) = 426(3) K, *J*_Dy–Dy_ = −4.3(1) mK, *J*_Er–Er_ = −1.1(3) mK, *g*_Dy_ = 1.38(1), *g*_Er_ = 1.22(1), δχ_0_(**1**) = 0.0035(2) emu mol^–1^, and δχ_0_(**2**) = 0.0033(2) emu mol^–1^. All parameters, except the coupling constants, are found to be essentially identical to the previous fit. This further demonstrates the robustness of the result and indicates that including or not including dipolar couplings does not affect our analysis. The values of the exchange coupling constants naturally differ, as in the former fit, they represent the combined effect of both exchange and dipolar couplings, whereas in this fit, they solely represent exchange couplings.

### Analysis Including Fe_2_Er_2_ (**3**) and Fe_2_Dy_2_ (**4**)

The actual goal is to achieve a simultaneous fit to all data for all four compounds, Al_2_Er_2_ (**1**), Al_2_Dy_2_ (**2**), Fe_2_Er_2_ (**3**), and Fe_2_Dy_2_ (**4**). However, since the numerical simulation of the powder magnetization curves was found to be time-consuming for Fe_2_Er_2_ (**3**) and Fe_2_Dy_2_ (**4**), the above fitting approach could not be simply carried over. Instead, least-squares fitting using the Levenberg–Marquardt algorithm was performed, where the χ*T* and *M*(*B*) curves of Al_2_Er_2_ (**1**) and Al_2_Dy_2_ (**2**) and the χ*T* curves of Fe_2_Er_2_ (**3**) and Fe_2_Dy_2_ (**4**) were included, but the *M*(*B*) curves of Fe_2_Er_2_ (**3**) and Fe_2_Dy_2_ (**4**) were excluded. The *M*(*B*) curves of Fe_2_Er_2_ (**3**) and Fe_2_Dy_2_ (**4**) were then retrospectively simulated for the parameters obtained in the fit and visually compared with the experimental data. The Levenberg–Marquardt fits were initialized using the results found above.

The fit model used again the AOM model for setting the bare ligand field parameters with the *e*_σ_ = *e*_σ_(*O*,*N*) and *e*_π,*c*_ = *e*_π,*s*_ = *e*_π_(*O*) parametrization for the bond strengths. The coupling parameters were *J*_Er–Er_(**1**), *J*_Er–Er_(**3**), *J*_Dy–Dy_(**2**), *J*_Dy–Dy_(**4**), *J*_Fe–Er_, *J*_Fe–Dy_, and *J*_Fe–Fe_(**3**), and *J*_Fe–Fe_(**4**). The Fe^III^–Fe^III^ coupling was found to be substantially different in the two compounds Fe_2_Er_2_ (**3**) and Fe_2_Dy_2_ (**4**) (vide infra) and was thus introduced through two separate parameters for each compound. The Landé *g*_Ln_ factors were fixed to their free ion values. The Fe^III^*g* factor was set to *g*_Fe_ = 1.95, which is slightly lower than the theoretically expected value of *g* = 2 for high-spin Fe^III^ ions but in the experimental range.^64,65^ The Fe^III^ single-ion anisotropy constants *D* and *E* were set to zero, *D* = *E* = 0. Since magnetic anisotropy of high-spin Fe^III^ is frequently observed with strengths |*D*| on the order 0.1 to 1 K,^64−66^ assuming zero anisotropy might seem unrealistic. However, the statement here is not that these parameters are zero but that from the available experimental data, they could not additionally be determined with statistical significance, i.e., setting them to values on the order of 1 K or to zero did not significantly affect the resulting fit curves and parameters. This was found to hold true even for the simplest possible case of the compound Fe_2_Y_2_ of the same family of butterflies, which represents a dimer of exchange coupled high-spin Fe^III^ ions.^56^ Fitting its magnetic data yielded *J*_Fe–Fe_ = −20.2(1) K and *g*_*Fe*_ = 1.94(1), but *D* and *E* could not be determined with significance. Therefore, not being able to determine *D* and *E* for Fe_2_Er_2_ (**3**) and Fe_2_Dy_2_ (**4**) is not due to too many parameters in the model but because the available powder data simply does not provide that information in the present cases.

The resulting best-fit parameters are compiled in Table 1 for the four compounds, and the resulting simulated curves are shown in Figures 2–4. In addition, the parameters *g*_Fe_ = 1.95, δχ_0_(**1**) = 0.0035 emu mol^–1^, δχ_0_(**2**) = 0.0034 emu mol^–1^, δχ_0_(**3**) = 0.0020 emu mol^–1^, and δχ_0_(**4**) = −0.0037 emu mol^–1^ were used. The fits to the χ*T* and *M*(*B*) data of Al_2_Er_2_ (**1**) and Al_2_Dy_2_ (**2**) and χ*T* data of Fe_2_Er_2_ (**3**) and Fe_2_Dy_2_ (**4**) are excellent (Figures 2 and 3). The simulated *M*(*B*) curves for Fe_2_Er_2_ (**3**) and Fe_2_Dy_2_ (**4**) (Figure 4) do not reproduce the data equally accurately but are still remarkably close to the data. Most importantly, the simulated curves reproduce the key features in the experimental data correctly, such as the crossing of the magnetization curves at ∼4.1 and ∼5.8 T for Fe_2_Er_2_ (**3**) and Fe_2_Dy_2_ (**4**), respectively. That is, the model captures the relevant physics remarkably well without the fit having seen that data.

**Table 1:** Best-Fit Parameters for the AOM Using *e*_σ_(*O,N*) and *e*_π_(*O)* Parametrization as Discussed in the Text^a^

	*J*_Ln–Ln_ [mK]	*J*_Fe–Ln_ [mK]	*J*_Fe–Fe_ [K]	*g*_Ln_	*e*_σ_(*O*, *N*) [K]	*e*_π_(*O*) [K]
Al_2_Dy_2_ (**2**)	0.9 (1)			1.3803 (3)	441 (5)	417 (5)
Al_2_Er_2_ (**1**)	0.0 (3)			1.2192 (5)		
Fe_2_Dy_2_ (**4**)	–17 (1)	120 (1)	–12.2 (2)	1.3803 (3)		
Fe_2_Er_2_ (**3**)	–23 (3)	–60 (3)	–18.7 (4)	1.2192 (5)		

a The resulting χ^2^ value is 2.81.

The AOM bond parameters were obtained as *e*_σ_(*O*, *N*) = 441(5) K and *e*_π_(*O*) = 417(5) K. These values are very close to those obtained from the above linked fits to the compounds Al_2_Er_2_ (**1**) and Al_2_Dy_2_ (**2**), which is a testimony of the robustness of the model, i.e., that the model and the employed parametrization are statistically significant. The *J*_Ln–Ln_ coupling constants come out with statistical significance, but except for Al_2_Dy_2_ (**2**), they should not be given scientific significance. This is because the values for the compounds Al_2_Er_2_ (**1**), Fe_2_Er_2_ (**3**), and Fe_2_Dy_2_ (**4**) are very small and fluctuate across different fits. Essentially, equally good fits could be obtained if the *J*_Ln–Ln_ constants were set to zero for compounds **1**, **3**, and **4**. The value of *J*_Ln–Ln_ for Al_2_Dy_2_ (**2**), however, was found to be relatively robust, which can be easily traced back to the fact that for this compound, it is associated with a clear feature in the magnetic data, namely, the upturn in χ*T* at low temperatures. A similar conclusion can be drawn for the values of *J*_Fe–Er_ and *J*_Fe–Dy_. While they come out with statistical significance, they fluctuate across different fits. These parameters are thus better described as lumped parameters that adjust the fit curves to reach slightly better matches to the data but bear little scientific relevance. The couplings *J*_Fe–Fe_ are determined to be weaker than that of the dimeric analogue Fe_2_Y_2_ and significantly different for the two compounds Fe_2_Er_2_ (**3**) and Fe_2_Dy_2_ (**4**). The magnitude of *J*_Fe–Fe_ is related to the field at which a crossing of the curves in the *M*(*B*) data occurs, which for Fe_2_Er_2_ (**3**) and Fe_2_Dy_2_ (**4**) occur at ca. 4.1 and 5.8 T, respectively. Indeed, the ca. 30% smaller crossing field in Fe_2_Dy_2_ (**4**) as compared to Fe_2_Er_2_ (**3**) correlates well with the ca. 40% smaller magnitude of *J*_Fe–Fe_. In summary, the parameters *J*_Dy–Dy_(**2**), *J*_Fe–Fe_(**3**), *J*_Fe–Fe_(**4**), *e*_σ_(*O,N*), and *e*_π_(*O*) are not only statistically but clearly also scientifically significant and constitute the main result of this work. The bare and common ligand field parameters Ω_*kq,j*_ and *B*_*kq,j*_ associated with the determined bond strengths are given in Tables S2 and S3.

## Conclusions

A surprisingly accurate and consistent modeling of the magnetism of four members of a 3d-4f butterfly family was achieved using only two parameters for describing the lanthanide ligand field in all four compounds. The employed methodology utilized a black box implementation of the AOM and disregarded any a priori physical or chemical motivation in the parameter selection. Instead, the transferability of AOM parameters across the four isostructural compounds was demanded. This led to a reduced parametrization consisting of two bond parameters *e*_σ_(*O*,*N*) and *e*_π_(*O*), which simultaneously characterize the susceptibility and magnetization data of the four compounds to a remarkable accuracy. To the best of the authors’ knowledge, such an efficient reduction in ligand field parameters for describing a broad set of thermodynamic data for low symmetry ligand field complexes, which included polynuclear members, is exceptional.

The quality of the experimental data was found to be crucial for achieving a well working modeling across the different compounds. This highlights the importance of a consistent measurement protocol and minimization of experimental inaccuracies for this kind of analysis. It is challenging to separate variations in the parameter values due to experimental inaccuracies from those arising from different physics of the compounds, and otherwise, this would significantly complicate an already overparametrized description, leading to widely different nonunique parameter sets.

The success of the reduced parametrization suggests that in the considered systems, the AOM is indeed capable of capturing the highly nonlinear correlations between the ligand field parameters *B*_*kq*_ with good precision. Interestingly, the best-performing parametrization of the bond strengths and its values obtained in this work aligns reasonably well with chemical expectations. That is, the experienced practitioner may choose them more carefully such as to more accurately capture the details of the chemical nature of the ligands, but both the parametrization and the values are in a reasonable ballpark. However, it is crucial to note that the nature of the employed approach does not require such assigned chemical meaning to be true. In fact, a high accuracy of a fit does not necessarily imply the validity of the resulting chemical or physical model. The transferability of the ligand field parameters implied by this work for the available magnetic data may thus be possibly contradicted by insights from more elaborate experiments. Nonetheless, the success of the proposed method suggests that setting aside prior chemical intuition and instead emphasizing transferability can be a more effective strategy for achieving reduced ligand field parametrizations, which are still consistent with the chemical nature of the ligands.

In addition, the success of the reduced parametrization also suggests that the information content in powder magnetic data about the lanthanide ligand field can be very low, in the present case, as low as only two free parameters, even when a relatively large data set consisting of both susceptibility and magnetization curves on four compounds is collected. While it is a priori clear, as discussed in the introduction, that this type of data has low information content, one may find this result nevertheless surprising. The finding should have implications for the interpretation of such experimental data. For instance, comparing the result of *ab initio* theory to a measured powder susceptibility curve may not be a strong confirmation of the theory. Also, parameter values obtained from fitting individual curves to multiparameter models may not be of scientific significance.

Extending the presented approach to other compound families will further illuminate the potential for chemical interpretability as a possible byproduct of the results. Furthermore, the framework and methodology used in this work may serve as a promising foundation for the reduction of ligand field parameters through approaches based on physics-informed machine learning as well.

## Supporting Information Available

Positions of the ligand atoms surrounding Ln_1_ and Ln_2_ (Table S1); ligand field parameters for the two lanthanide ions in the AOM *e*_σ_(*O,N*), *e*_π_(*O*) parametrization (Table S2); standardized ligand field parameters for the two lanthanide ions in the AOM *e*_σ_(*O,N*), *e*_π_(*O*) parametrization (Table S3); sketch of the 4f orbitals and the associated AOM bonding types and parameters (Figure S1); χ^2^ plots for the model assuming rhombic anisotropy for the lanthanides (Figure S2); χ^2^ plots for the PCM using the *q*(*O*), *q*(N*)* parametrization (Figure S3); and χ^2^ plots for the AOM using the *e*_σ_(*O*), *e*_σ_(*N)* parametrization (Figure S4) (PDF)

## References

[ref1] Luis F.; Repollés A.; Martínez-Pérez M. J.; Aguilà D.; Roubeau O.; Zueco D.; Alonso P. J.; Evangelisti M.; Camón A.; Sesé J.; Barrios L. A.; Aromí G. Molecular prototypes for spin-based CNOT and SWAP quantum gates. Phys. Rev. Lett. 2011, 107, 117203 10.1103/PhysRevLett.107.117203. 22026699

[ref2] Bernot K.; Daiguebonne C.; Calvez G.; Suffren Y.; Guillou O. A Journey in Lanthanide Coordination Chemistry: From Evaporable Dimers to Magnetic Materials and Luminescent Devices. Acc. Chem. Res. 2021, 54, 427–440. 10.1021/acs.accounts.0c00684. 33395256

[ref3] Gabarró-Riera G.; Aromí G.; Sañudo E. C. Magnetic molecules on surfaces: SMMs and beyond. Coord. Chem. Rev. 2023, 475, 214858 10.1016/j.ccr.2022.214858.

[ref4] Chiesa A.; Santini P.; Garlatti E.; Luis F.; Carretta S. Molecular nanomagnets: a viable path toward quantum information processing?. Rep. Prog. Phys. 2024, 87, 034501 10.1088/1361-6633/ad1f81. 38314645

[ref5] Chicco S.; Allodi G.; Chiesa A.; Garlatti E.; Buch C. D.; Santini P.; de Renzi R.; Piligkos S.; Carretta S. Proof-of-Concept Quantum Simulator Based on Molecular Spin Qudits. J. Am. Chem. Soc. 2024, 146, 1053–1061. 10.1021/jacs.3c12008. 38147824 PMC10785809

[ref6] Leuenberger M. N.; Loss D. Quantum computing in molecular magnets. Nature 2001, 410, 789–793. 10.1038/35071024. 11298441

[ref7] Moreno-Pineda E.; Godfrin C.; Balestro F.; Wernsdorfer W.; Ruben M. Molecular spin qudits for quantum algorithms. Chem. Soc. Rev. 2018, 47, 501–513. 10.1039/C5CS00933B. 29147698

[ref8] Gaita-Ariño A.; Luis F.; Hill S.; Coronado E. Molecular spins for quantum computation. Nat. Chem. 2019, 11, 301–309. 10.1038/s41557-019-0232-y. 30903036

[ref9] Biard H.; Moreno-Pineda E.; Ruben M.; Bonet E.; Wernsdorfer W.; Balestro F. Increasing the Hilbert space dimension using a single coupled molecular spin. Nat. Commun. 2021, 12, 4443 10.1038/s41467-021-24693-6. 34290250 PMC8295329

[ref10] Sessoli R. Toward the Quantum Computer: Magnetic Molecules Back in the Race. ACS Cent. Sci. 2015, 1, 473–474. 10.1021/acscentsci.5b00384. 27163011 PMC4827668

[ref11] Jensen J.; Mackintosh A. Rare Earth Magnetism: Structures and Excitations, International Series of Monographs on Physics 81; Clarendon Press: Oxford, 1991.

[ref12] Woodruff D. N.; Winpenny R. E. P.; Layfield R. A. Lanthanide single-molecule magnets. Chem. Rev. 2013, 113, 5110–5148. 10.1021/cr400018q. 23550940

[ref13] Luzon J.; Sessoli R. Lanthanides in molecular magnetism: so fascinating, so challenging. Dalton Trans. 2012, 41, 13556–13567. 10.1039/c2dt31388j. 22936346

[ref14] Zabala-Lekuona A.; Seco J. M.; Colacio E. Single-Molecule Magnets: From Mn_12_-ac to dysprosium metallocenes, a travel in time. Coord. Chem. Rev. 2021, 441, 213984 10.1016/j.ccr.2021.213984.

[ref15] Bernot K. Get under the Umbrella: A Comprehensive Gateway for Researchers on Lanthanide-Based Single-Molecule Magnets. Eur. J. Inorg. Chem. 2023, 26, e202300336 10.1002/ejic.202300336.

[ref16] Benelli C.; Gatteschi D. Introduction to Molecular Magnetism: From Transition Metals to Lanthanides; Wiley-VCH: Weinheim, 2015.

[ref17] Rinehart J. D.; Long J. R. Exploiting single-ion anisotropy in the design of f-element single-molecule magnets. Chem. Sci. 2011, 2, 2078–2085. 10.1039/c1sc00513h.

[ref18] Sessoli R.; Powell A. K. Strategies towards single molecule magnets based on lanthanide ions. Coord. Chem. Rev. 2009, 253, 2328–2341. 10.1016/j.ccr.2008.12.014.

[ref19] Ishikawa N. Single molecule magnet with single lanthanide ion. Polyhedron 2007, 26, 2147–2153. 10.1016/j.poly.2006.10.022.

[ref20] Newman D. J. Theory of lanthanide crystal fields. Adv. Phys. 1971, 20, 197–256. 10.1080/00018737100101241.

[ref21] Hutchings M. T. Point-Charge Calculations of Energy Levels of Magnetic Ions in Crystalline Electric Fields. In Solid State Physics; Elsevier, 1964; Vol. 16, pp 227–273.

[ref22] Layfield R. A.; Murugesu M. Lanthanides and Actinides in Molecular Magnetism; Wiley, 2015.

[ref23] Suta M.; Cimpoesu F.; Urland W. The angular overlap model of ligand field theory for f elements: An intuitive approach building bridges between theory and experiment. Coord. Chem. Rev. 2021, 441, 213981 10.1016/j.ccr.2021.213981.

[ref24] Malkin B. Z. Crystal field and Electron–Phonon Interaction in Rare-Earth Ionic Paramagnets. In Spectroscopy of Solids Containing Rare Earth Ions, Modern Problems in Condensed Matter Sciences; Elsevier, 1987; pp 13–50.

[ref25] Baldoví J. J.; Clemente-Juan J. M.; Coronado E.; Gaita-Ariño A.; Palii A. An updated version of the computational package SIMPRE that uses the standard conventions for Stevens crystal field parameters. J. Comput. Chem. 2014, 35, 1930–1934. 10.1002/jcc.23699. 25087575

[ref26] Racah G. Theory of Complex Spectra. II. Phys. Rev. 1942, 62, 438–462. 10.1103/PhysRev.62.438.

[ref27] Tondello E.; Michelis G. de.; Oleari L.; Di Sipio L. Slater-condon parameters for atoms and ions of the first transition period. Coord. Chem. Rev. 1967, 2, 53–63. 10.1016/S0010-8545(00)80194-0.

[ref28] Goodenough J. B. Spin-Orbit-Coupling Effects in Transition-Metal Compounds. Phys. Rev. 1968, 171, 466–479. 10.1103/PhysRev.171.466.

[ref29] Cole G. M.; Garrett B. B. Atomic and molecular spin-orbit coupling constants for 3d transition metal ions. Inorg. Chem. 1970, 9, 1898–1902. 10.1021/ic50090a020.

[ref30] Gschneidner K. A. Jr; Eyring L. Handbook on the Physics and Chemistry of Rare Earths 1996.

[ref31] Ungur L.; Chibotaru L. F. Ab Initio Crystal Field for Lanthanides. Chem. - Eur. J. 2017, 23, 3708–3718. 10.1002/chem.201605102. 27983776

[ref32] Hallmen P. P.; Köppl C.; Rauhut G.; Stoll H.; van Slageren J. Fast and reliable ab initio calculation of crystal field splittings in lanthanide complexes. J. Chem. Phys. 2017, 147, 164101 10.1063/1.4998815. 29096514

[ref33] Lunghi A.; Totti F. The Role of Anisotropic Exchange in Single Molecule Magnets: A CASSCF/NEVPT2 Study of the Fe_4_ SMM Building Block [Fe_2_(OCH_3_)_2_(dbm)_4_] Dimer. Inorganics 2016, 4, 28 10.3390/inorganics4040028.

[ref34] Reta D.; Kragskow J. G. C.; Chilton N. F. Ab Initio Prediction of High-Temperature Magnetic Relaxation Rates in Single-Molecule Magnets. J. Am. Chem. Soc. 2021, 143, 5943–5950. 10.1021/jacs.1c01410. 33822599

[ref35] Vonci M.; Giansiracusa M. J.; van den Heuvel W.; Gable R. W.; Moubaraki B.; Murray K. S.; Yu D.; Mole R. A.; Soncini A.; Boskovic C. Magnetic Excitations in Polyoxotungstate-Supported Lanthanoid Single-Molecule Magnets: An Inelastic Neutron Scattering and ab Initio Study. Inorg. Chem. 2017, 56, 378–394. 10.1021/acs.inorgchem.6b02312. 27977150

[ref36] Baldoví J. J.; Borrás-Almenar J. J.; Clemente-Juan J. M.; Coronado E.; Gaita-Ariño A. Modeling the properties of lanthanoid single-ion magnets using an effective point-charge approach. Dalton Trans. 2012, 41, 13705–13710. 10.1039/c2dt31411h. 22961120

[ref37] Liu F.; Krylov D. S.; Spree L.; Avdoshenko S. M.; Samoylova N. A.; Rosenkranz M.; Kostanyan A.; Greber T.; Wolter A. U. B.; Büchner B.; Popov A. A. Single molecule magnet with an unpaired electron trapped between two lanthanide ions inside a fullerene. Nat. Commun. 2017, 8, 16098 10.1038/ncomms16098. 28706223 PMC5519982

[ref38] Urland W. On the ligand-field potential for f electrons in the angular overlap model. Chem. Phys. 1976, 14, 393–401. 10.1016/0301-0104(76)80136-X.

[ref39] Bazhenova T. A.; Yakushev I. A.; Lyssenko K. A.; Maximova O. V.; Mironov V. S.; Manakin Y. V.; Kornev A. B.; Vasiliev A. N.; Yagubskii E. B. Ten-Coordinate Lanthanide [Ln(HL)(L)] Complexes (Ln = Dy, Ho, Er, Tb) with Pentadentate N_3_O_2_-Type Schiff-Base Ligands: Synthesis, Structure and Magnetism. Magnetochemistry 2020, 6, 60 10.3390/magnetochemistry6040060.

[ref40] Reu O. S.; Palii A. V.; Ostrovsky S. M.; Tregenna-Piggott P. L. W.; Klokishner S. I. A model of magnetic and relaxation properties of the mononuclear Pc2Tb(−)TBA+ complex. Inorg. Chem. 2012, 51, 10955–10965. 10.1021/ic3014078. 23013596

[ref41] Jung J.; Islam M. A.; Pecoraro V. L.; Mallah T.; Berthon C.; Bolvin H. Derivation of Lanthanide Series Crystal Field Parameters From First Principles. Chem. - Eur. J. 2019, 25, 15112–15122. 10.1002/chem.201903141. 31496013

[ref42] Baldoví J. J.; Clemente-Juan J. M.; Coronado E.; Gaita-Ariño A. Two pyrazolylborate dysprosium(III) and neodymium(III) single ion magnets modeled by a Radial Effective Charge approach. Polyhedron 2013, 66, 39–42. 10.1016/j.poly.2013.01.034.

[ref43] Gajek Z. Enhanced angular overlap model for nonmetallic f -electron systems. Phys. Rev. B 2005, 72, 045139 10.1103/PhysRevB.72.045139.

[ref44] Buchhorn M.; Krewald V. AOMadillo: A program for fitting angular overlap model parameters. J. Comput. Chem. 2024, 45, 122–134. 10.1002/jcc.27224. 37767988

[ref45] Bronova A.; Bredow T.; Glaum R.; Riley M. J.; Urland W. BonnMag: Computer program for ligand-field analysis of f^n^ systems within the angular overlap model. J. Comput. Chem. 2018, 39, 176–186. 10.1002/jcc.25096. 29143342

[ref46] Rakitin Y. V.; Rakitina V. M.; Kalinnikov V. T. Calculation of the Magnetic Properties of d and f Transition Metal Complexes in Terms of the Angular Overlap Model. Russ. J. Coord. Chem. 2005, 31, 477–482. 10.1007/s11173-005-0122-2.

[ref47] Hoggard P. E. Angular Overlap Model Parameters. In Optical Spectra and Chemical Bonding in Inorganic Compounds; Springer, 2004; Vol. 106, pp 37–57.

[ref48] Bertini I.; Gatteschi D.; Scozzafava A. Ligand field interpretation of high-spin trigonal-bipyramidal cobalt(II) complexes. Inorg. Chem. 1975, 14, 812–815. 10.1021/ic50146a024.

[ref49] Maity S.; Mondal A.; Konar S.; Ghosh A. The role of 3d-4f exchange interaction in SMM behaviour and magnetic refrigeration of carbonato bridged CuLn (Ln = Dy, Tb and Gd) complexes of an unsymmetrical N_2_O_4_ donor ligand. Dalton Trans. 2019, 48, 15170–15183. 10.1039/C9DT02627D. 31565726

[ref50] Peng Y.; Powell A. K. What do 3d-4f butterflies tell us?. Coord. Chem. Rev. 2021, 426, 213490 10.1016/j.ccr.2020.213490.

[ref51] Stevens K. W. H. Matrix Elements and Operator Equivalents Connected with the Magnetic Properties of Rare Earth Ions. Proc. Phys. Soc. A 1952, 65, 209 10.1088/0370-1298/65/3/308.

[ref52] Ishikawa N.; Sugita M.; Okubo T.; Tanaka N.; Iino T.; Kaizu Y. Determination of ligand-field parameters and f-electronic structures of double-decker bis(phthalocyaninato)lanthanide complexes. Inorg. Chem. 2003, 42, 2440–2446. 10.1021/ic026295u. 12665381

[ref53] Ishikawa N. Simultaneous Determination of Ligand-Field Parameters of Isostructural Lanthanide Complexes by Multidimensional Optimization. J. Phys. Chem. A 2003, 107, 5831–5835. 10.1021/jp034433a.

[ref54] Hiller M.; Krieg S.; Ishikawa N.; Enders M. Ligand-Field Energy Splitting in Lanthanide-Based Single-Molecule Magnets by NMR Spectroscopy. Inorg. Chem. 2017, 56, 15285–15294. 10.1021/acs.inorgchem.7b02704. 29200279

[ref55] Pavlov A. A. Paramagnetic NMR Spectroscopy as a Tool for Studying the Electronic Structures of Lanthanide and Transition Metal Complexes. INEOS Open 2020, 2, 153–162. 10.32931/io1922r.

[ref56] Peng Y.; Mereacre V.; Anson C. E.; Powell A. K. Multiple superhyperfine fields in a {DyFe_2_Dy} coordination cluster revealed using bulk susceptibility and (57)Fe Mössbauer studies. Phys. Chem. Chem. Phys. 2016, 18, 21469–21480. 10.1039/C6CP02942F. 27424877

[ref57] Peng Y.; Mereacre V.; Anson C. E.; Powell A. K. Butterfly M_2_^III^Er_2_ (M^III^ = Fe and Al) SMMs: Synthesis, Characterization, and Magnetic Properties. ACS Omega 2018, 3, 6360–6368. 10.1021/acsomega.8b00550. 31458819 PMC6644375

[ref58] Tinkham M. Group Theory and Quantum Mechanics; Dover Publicatiion: New York, 2003.

[ref59] Waldmann O. Symmetry and energy spectrum of high-nuclearity spin clusters. Phys. Rev. B 2000, 61, 6138–6144. 10.1103/PhysRevB.61.6138.

[ref60] Schäffer C. E.; Jørgensen C. K. The angular overlap model, an attempt to revive the ligand field approaches. Mol. Phys. 1965, 9, 401–412. 10.1080/00268976500100551.

[ref61] McHugh M. L. The chi-square test of independence. Biochem. Med. 2013, 23, 143–149. 10.11613/BM.2013.018. PMC3900058 23894860

[ref62] Press W. H.; Flannery B. P.; Teukolsky S. A. Numerical Recipes. The Art of Scientific Computing; University Press: Cambridge, 1986.

[ref63] Mayer J.; Nugent W. A. Metal-Ligand Multiple Bonds: The Chemistry of Transition Metal Complexes Containing Oxo, Nitrido, Imido, Alkylidene, or Alkylidyne Ligands; Wiley, 1988.

[ref64] Waldmann O.; Koch R.; Schromm S.; Schülein J.; Müller P.; Bernt I.; Saalfrank R. W.; Hampel F.; Balthes E. Magnetic Anisotropy of a Cyclic Octanuclear Fe(III) Cluster and Magneto-Structural Correlations in Molecular Ferric Wheels. Inorg. Chem. 2001, 40, 2986–2995. 10.1021/ic0012827. 11399165

[ref65] Ummethum J.; Nehrkorn J.; Mukherjee S.; Ivanov N. B.; Stuiber S.; Strässle T.; Tregenna-Piggott P. L. W.; Mutka H.; Christou G.; Waldmann O.; Schnack J. Discrete antiferromagnetic spin-wave excitations in the giant ferric wheel Fe_18_. Phys. Rev. B 2012, 86, 104403 10.1103/PhysRevB.86.104403.

[ref66] Dreiser J.; Waldmann O.; Carver G.; Dobe C.; Güdel H.-U.; Weihe H.; Barra A.-L. High-Frequency Electron-Spin-Resonance Study of the Octanuclear Ferric Wheel CsFe_8_. Inorg. Chem. 2010, 49, 8729–8735. 10.1021/ic100664g. 20831210

