# Supplementary material for: Transferability of Ligand Field Parameters in a Family of 3d-4f M2Ln2 Butterfly Single-Molecule Magnets
Source: Inorg Chem. 2025 Mar 17;64(12):6115–24. doi: 10.1021/acs.inorgchem.4c05421 (PMC11962824; doi:10.1021/acs.inorgchem.4c05421)
Supplement: Supplementary file 1 — ic4c05421_si_001.pdf [file ic4c05421_si_001.pdf]

SUPPLEMENTARY INFORMATION

Transferability of Ligand Field Parameters in a Family of 3d-4f M_2Ln_2 Butterfly Single Molecule Magnets

Julius Mutschler[1], Thomas Ruppert[2], Julius Strahringer[1], Sören Schlittenhardt[3], Zayan Ahsan Ali[1], Yan Peng[2†], Christopher E. Anson[2], Mario Ruben[3,4], Annie K. Powell[2,3,5], Oliver Waldmann[1]**

[1] Physikalisches Institut, Universität Freiburg, Hermann-Herder-Strasse 3, 79104 Freiburg, Germany.

[2] Institute of Inorganic Chemistry, Karlsruhe Institute of Technology, Kaiserstr. 12, 76131 Karlsruhe, Germany.

[3] Institute of Nanotechnology, Karlsruhe Institute of Technology, Kaiserstr. 12, 76131 Karlsruhe, Germany.

[4] Centre Européen de Sciences Quantiques (CESQ), Institut de Science et d'Ingénierie Supramoléculaires (ISIS), 8 allée Gaspard Monge, BP 70028, 67083, Strasbourg Cedex, France

[5] Institute of Quantum Materials and Technologies, Karlsruhe Institute of Technology, Kaiserstr. 12, 76131 Karlsruhe, Germany.

Corresponding Author's Email: oliver.waldmann@physik.uni-freiburg.de

Corresponding Author's Email: annie.powell@kit.edu

SUPPLEMENTARY INFORMATION

Content:

- Table S1: Positions of the ligand atoms surrounding Ln_1 and Ln_2.

- Table S2: Ligand field parameters for the two lanthanide ions in the AOM $e_\sigma(O,N)$, $e_\pi(O)$ parametrization.

- Table S3: Standardized ligand field parameters for the two lanthanide ions in the AOM $e_\sigma(O,N)$, $e_\pi(O)$ parametrization.

- Figure S1: Sketch of the 4f orbitals and the associated AOM bonding types and parameters.

- Figure S2: chi^2 plots for the model assuming rhombic anisotropy for the lanthanides.

- Figure S3: chi^2 plots for the PCM using the $q(O)$, $q(N)$ parametrization.

- Figure S4: chi^2 plots for the AOM using the $e_\sigma(O)$, $e_\sigma(N)$ parametrization.

Table S1: Positions of the ligand atoms surrounding Ln_1 and Ln_2.

		LN_1				LN_2		
**Ion**		**distance**	**theta**	**phi**		**distance**	**theta**	**phi**
O1		2.421564	1.175687	1.580861		2.421564	1.965906	-1.56073
O2		2.320102	2.028631	0.788371		2.320123	1.112957	-2.35327
O3		2.33082	2.023287	2.370949		2.330739	1.11828	-0.77065
O5		2.421173	0.897385	0.110284		2.421173	2.244208	-3.03131
O7		2.404476	0.941128	3.038978		2.404476	2.200465	-0.10261
O8		2.437004	1.706138	-2.18168		2.43701	1.435446	0.95995
O9		2.453791	0.922143	-1.62178		2.453816	2.219432	1.519855
N1		2.602044	3.030624	-1.70852		2.602044	0.110969	1.433074
N2		2.564055	1.939124	-0.8293		2.564055	1.202468	2.312289

Table S2: Bare ligand field parameters Ω_{kq} and ligand field parameters B_{kq} for the two lanthanide ions in the parametrization $e_\sigma(O,N)$ and $e_\pi(O)$ discussed in the main text. The employed ligand positions are given in Table SI1.

k, q	Ω_{kq}(Ln1) [K]	Ω_{kq}(Ln2) [K]	B_{kq}(Dy1) [10^{-k}K]	B_{kq}(Dy2) [10^{-k}K]	B_{kq}(Er1) [10^{-k}K]	B_{kq}(Er2) [10^{-k}K]
2, 0	-281.31	-281.30	178.61	178.61	-71.44	-71.44
2, 1	-58.49	-58.45	37.14	37.11	-14.85	-14.85
2, 2	-343.08	-343.10	217.83	217.84	-87.13	-87.14
4, 0	-64.80	-64.81	38.36	38.37	-28.77	-28.78
4, 1	153.24	153.26	-90.72	-90.73	68.04	68.05
4 ,2	274.86	274.89	-162.72	-162.74	122.04	122.05
4, 3	338.41	338.12	-200.34	-200.17	150.26	150.13
4, 4	-309.56	-309.49	183.26	183.22	-137.44	-137.41
6, 0	32.05	32.04	33.17	33.16	66.33	66.33
6, 1	-100.24	-100.23	-103.75	-103.73	-207.49	-207.46
6, 2	-12.04	-12.04	-12.46	-12.46	-24.92	-24.91
6, 3	-389.07	-389.06	-402.67	-402.67	-805.34	-805.33
6, 4	-310.79	-310.77	-321.65	-321.63	-643.31	-643.27
6, 5	768.17	767.96	795.03	794.81	1590.06	1589.62
6, 6	13.80	13.81	14.28	14.29	28.57	28.59
2, -1	-732.33	-732.30	464.97	464.95	-185.99	-185.98
2, -2	347.32	347.28	-220.52	-220.49	88.21	88.20
4, -3	1.81	1.80	-1.07	-1.07	0.80	0.80
4, -4	-132.17	-132.20	78.25	78.26	-58.68	-58.70
4, -5	-323.85	-323.97	191.72	191.79	-143.79	-143.84
4, -6	-256.65	-256.62	151.94	151.92	-113.95	-113.94
6, -1	191.16	191.15	197.84	197.83	395.68	395.66
6, -2	81.57	81.57	84.43	84.42	168.85	168.85
6, -3	-533.68	-533.66	-552.34	-552.32	-1104.67	-1104.65
6, -4	-10.25	-10.29	-10.61	-10.65	-21.22	-21.29
6, -5	-1844.66	-1844.54	-1909.16	-1909.04	-3818.31	-3818.07
6, -6	303.91	303.89	314.54	314.52	629.07	629.03

Table S3: Standardized bare ligand field parameters Ω_{kq} and ligand field parameters B_{kq} for the two lanthanide ions in the parametrization $e_\sigma(O,N)$ and $e_\pi(O)$ discussed in the main text. The standardization of these parameters consists of rotating the ligand field parameters given in Table SI2 by the Euler angles $\theta = 76.064°$, $\varphi = 77.022°$ and $\alpha = -68.851°$ for Ln_1 and $\theta = 76.065°$, $\varphi = 77.023°$ and $\alpha = -68.854°$ for Ln_2., which make the parameters for $k = 2$ with $q = 1, -1, -2$ zero.

k, q	$\Omega_{kq}(Ln_1)$ [K]	$\Omega_{kq}(Ln_2)$ [K]	$B_{kq}(Dy_1)$ [10^{-k}K]	$B_{kq}(Dy_2)$ [10^{-k}K]	$B_{kq}(Er_1)$ [10^{-k}K]	$B_{kq}(Er_2)$ [10^{-k}K]
2, 0	428.09	428.07	-271.80	-271.79	108.72	108.72
2, 1	0.00	0.00	0.00	0.00	0.00	0.00
2, 2	246.79	246.78	-156.69	-156.69	62.67	62.67
4, 0	43.05	43.04	-25.49	-25.48	19.12	19.11
4, 1	-377.28	-377.28	223.36	223.35	-167.51	-167.51
4 ,2	-109.38	-109.34	64.75	64.73	-48.57	-48.55
4, 3	669.12	669.19	-396.10	-396.17	297.10	297.12
4, 4	-391.47	-391.51	231.76	231.79	-173.79	-173.85
6, 0	9.91	9.92	10.26	10.27	20.52	20.54
6, 1	400.45	400.40	414.45	414.40	828.87	828.80
6, 2	-33.17	-33.18	-34.33	-34.34	-68.66	-68.68
6, 3	597.02	596.92	617.88	617.77	1235.81	1235.61
6, 4	-130.73	-130.79	-135.29	-135.37	-270.54	-270.72
6, 5	-1582.20	-1582.21	-1637.55	-1637.64	-3274.76	-3274.98
6, 6	-163.33	-163.24	-169.06	-169.00	-337.84	-337.94
2, -1	0.00	0.00	0.00	0.00	0.00	0.00
2, -2	0.00	0.00	0.00	0.00	0.00	0.00
4, -3	37.11	37.04	-21.96	-21.94	16.48	16.46
4, -4	-37.66	-37.67	22.29	22.31	-16.72	-16.72
4, -5	295.04	295.32	-174.67	-174.85	130.95	131.17
4, -6	292.02	292.08	-172.87	-172.90	129.68	129.69
6, -1	-110.46	-110.45	-114.32	-114.31	-228.72	-228.59
6, -2	-14.21	-14.14	-14.70	-14.63	-29.43	-29.27
6, -3	274.16	274.01	283.76	283.63	567.30	567.27
6, -4	18.87	18.86	19.52	19.51	39.08	39.05
6, -5	964.11	964.19	997.77	997.72	1996.23	1995.77
6, -6	454.47	454.40	470.35	470.26	940.75	940.61

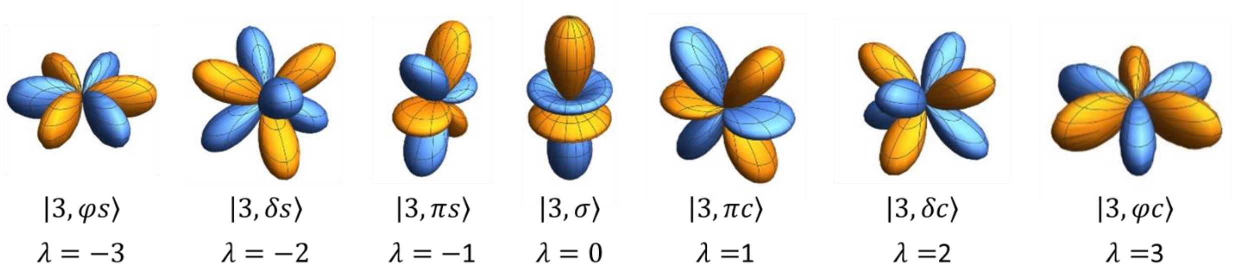

Figure S1: The seven 4f orbitals, and the associated bonding type and bonding parameter in the AOM.

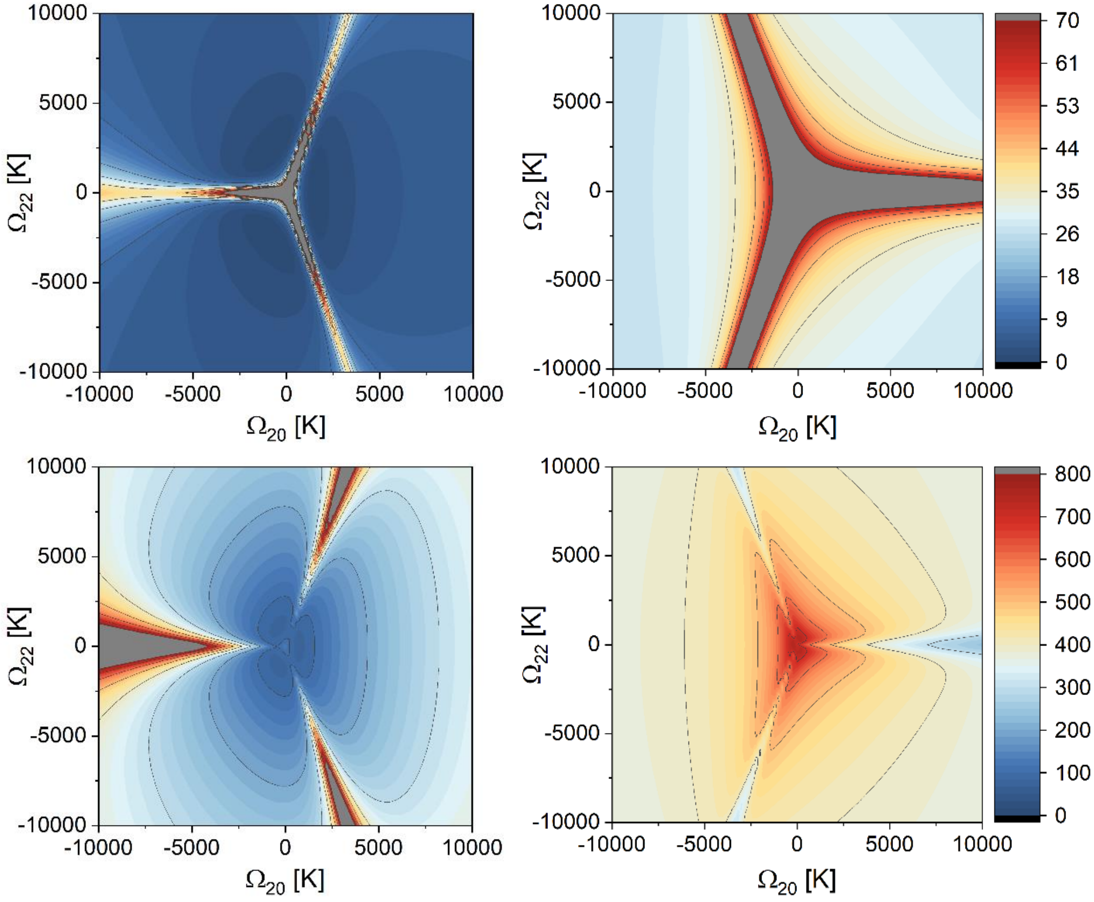

Figure S2: chi^2 plots for the model assuming rhombic anisotropy for the lanthanides (see main text). The coupling constants are J_{Dy-Dy} = 0 mK and J_{Er-Er} = -50 mK. Top left: $chi_M^2(\mathbf{2})$; Top right: $chi_M^2(\mathbf{1})$; Bottom left: $chi_{\chi T}^2(\mathbf{2})$; Bottom right: $chi_{\chi T}^2(\mathbf{1})$. The colour maps are from blue to red with range [0, 70] for the two magnetization grids and range [0, 800] for the two susceptibility grids.

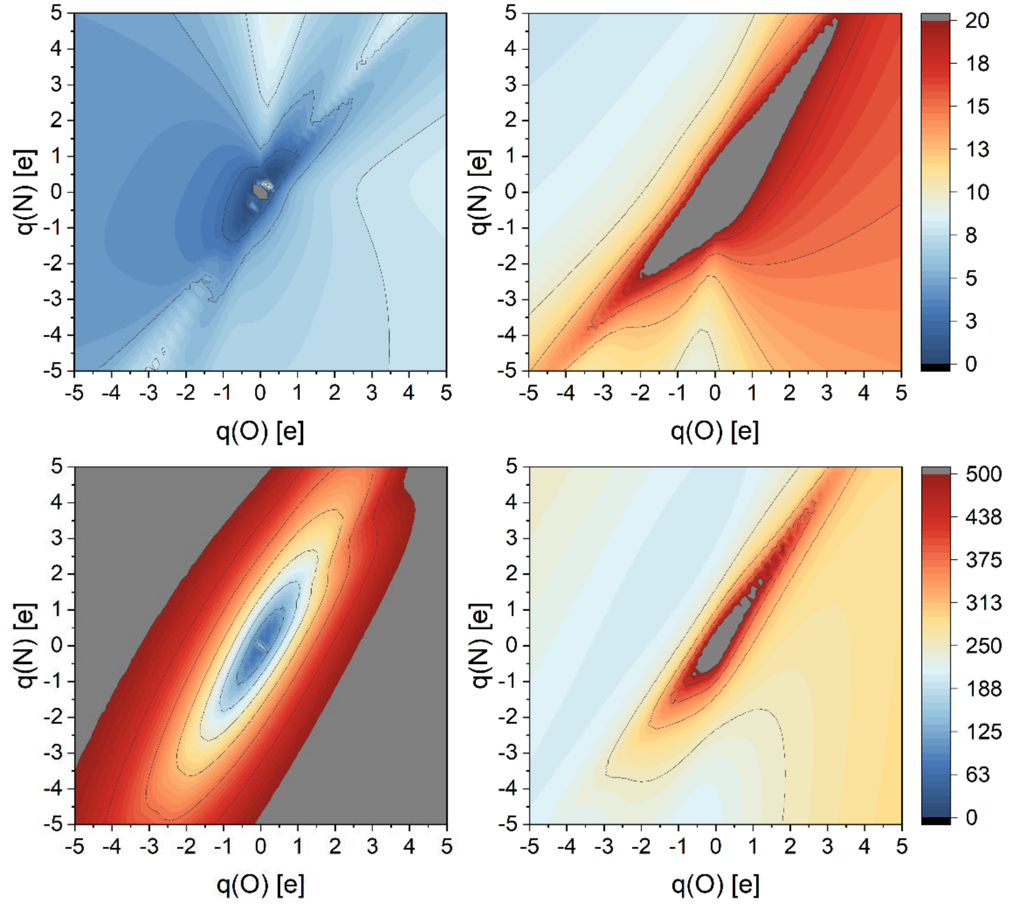

Figure S3: chi^2 plots for the PCM using the $q(O)$ and q(N) parametrization for the charges (see main text). The coupling constants are J_{Dy-Dy} = 3 mK and J_{Er-Er} = -20 mK. Top left: $chi^2_M(\mathbf{2})$; Top right: $chi^2_M(\mathbf{1})$; Bottom left: $chi^2_{\chi T}(\mathbf{2})$; Bottom right: $chi^2_{\chi T}(\mathbf{1})$. The colour maps are from blue to red with range [0, 20] for the two magnetization grids and range [0, 500] for the two susceptibility grids.

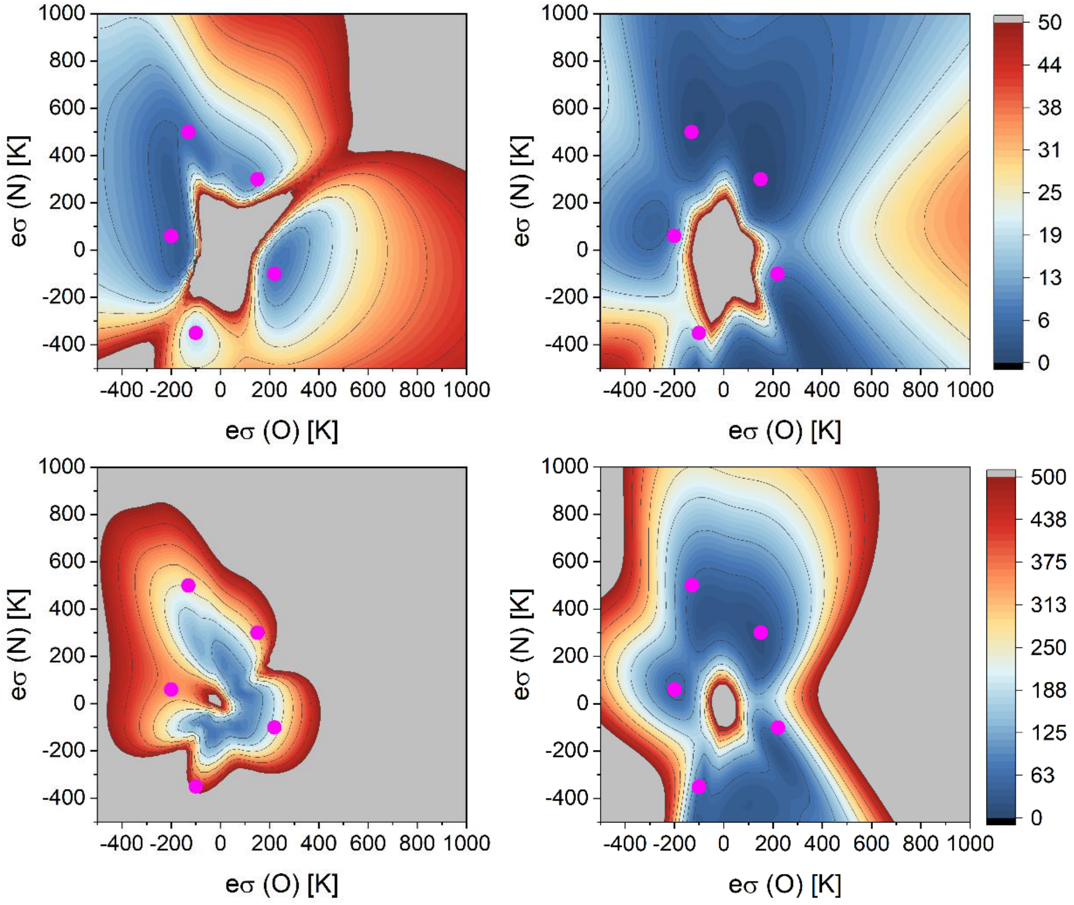

Figure S4: chi^2 plots for the AOM using the $e_\sigma(O)$ and $e_\sigma(N)$ parametrization for the bond strengths (see main text). The coupling constants are J_{Dy-Dy} = 40 mK and J_{Er-Er} = 0 mK. Top left: $chi_M^2(\mathbf{2})$; Top right: $chi_M^2(\mathbf{1})$; Bottom left: $chi_{\chi T}^2(\mathbf{2})$; Bottom right: $chi_{\chi T}^2(\mathbf{1})$. The colour maps are from blue to red with range [0, 50] for the two magnetization grids and range [0, 500] for the two susceptibility grids. The pink dots mark the five local minima.
